# Association Between Cesarean Scar and Pelvic Floor Muscle Tone at 6–8 Weeks Postpartum

**DOI:** 10.1007/s00192-024-06023-8

**Published:** 2025-01-09

**Authors:** Li Xiao, Nan Huang, Yanbiao Zhong, Yun Luo, Maoyuan Wang

**Affiliations:** 1 Department of Rehabilitation Medicine, First Affiliated Hospital of Gannan Medical University, No. 128 Jinling Road, Zhanggong District, Ganzhou City, 341000 Jiangxi Province China; 2 Ganzhou Key Laboratory of Rehabilitation Medicine, Ganzhou City, Jiangxi Province China; 3 Ganzhou Intelligent Rehabilitation Technology Innovation Center, Ganzhou City, Jiangxi Province China; 4 School of Rehabilitation Medicine, Gannan Medical University, Ganzhou City, Jiangxi Province China

**Keywords:** Cesarean section scar, Active pelvic floor muscle tone, Postpartum, Glazer protocol, Linear regression

## Abstract

**Introduction and Hypothesis:**

The relationship between cesarean section scars and active pelvic floor muscle tone lacks sufficient evidence. This study is aimed at investigating the relationship between the severity of cesarean section scars and active pelvic floor muscle tone in postpartum women.

**Methods:**

We conducted a prospective cross-sectional study of 604 women at 6–8 weeks postpartum. Active pelvic floor muscle tone was assessed using the Glazer protocol, and scar severity was categorized as no scar, normal scar, and hypertrophic scar. We collected data on demographic and clinical variables, including age, body mass index (BMI), and comorbidities. Linear regression analysis was employed to assess the association between scar severity and active pelvic floor muscle tone, adjusting for potential confounders.

**Results:**

Compared with the no scar group, the normal scar group exhibited higher levels of active pelvic floor muscle tone (β = 1.68 and 1.47), and the hypertrophic scar group had the highest levels of active pelvic floor muscle tone (β = 5.09 and 5.03). Active pelvic floor muscle tone was significantly higher in women with scars than in those without scars. The association remained significant after adjusting for age, BMI, and comorbidities. Moreover, women with hypertrophic scars exhibited higher active pelvic floor muscle tone than those with normal scars.

**Conclusions:**

Cesarean section scar severity is positively associated with increased active pelvic floor muscle tone in postpartum women. This finding highlights the importance of scar management and targeted pelvic floor rehabilitation to optimize postpartum recovery.

**Supplementary Information:**

The online version contains supplementary material available at 10.1007/s00192-024-06023-8.

## Introduction

Cesarean section is a common surgical procedure in obstetrics. However, it is associated with pelvic pain in postpartum women, particularly with chronic pelvic pain [1–3]. Cesarean section frequently results in adhesions that fix the uterus to the abdominal wall, and the tension generated by these adhesions is a significant factor in the development of severe chronic pelvic pain [4].

The use of surface electromyography (sEMG) for evaluating pelvic floor dysfunction is regarded as an objective, non-invasive diagnostic method. Additionally, it is convenient, cost effective, and easily manageable by medical personnel [5]. Surface electrodes can detect changes in muscle tension to measure active muscle tone, and these measurements can be validated through readings on the display or audio feedback [6]. The Glazer protocol, a widely utilized standardized approach to assessing pelvic floor muscle activity using electromyography, encompasses pre-baseline rest, rapid contraction, sustained contraction, endurance contraction, and post-baseline rest [7, 8]. Therefore, in this study, we assessed the active tension of the pelvic floor muscles using the measurements of pre-baseline rest and post-baseline rest.

Skin scars can be categorized as normal (physiological) or abnormal (pathological), with the latter primarily encompassing hypertrophic scars and keloids [9, 10]. Hyperplastic scars are firm, raised at the site of the injury, and may cause symptoms. They typically develop within 4 to 8 weeks of the injury and frequently occur in extensor joints and other areas of high tension [11]. The appearance of hypertrophic scars and keloids not only increases the psychological burden on patients, but severe scar contracture also impairs patients' physical function, making it difficult for them to live normally [12].

At present, the available evidence regarding the link between cesarean section scar and active pelvic floor muscle tone is inadequate. To address this knowledge gap, our primary objective is to assess the correlation between cesarean section scars and active pelvic floor muscle tone, as well as the potential implications of this relationship.

## Materials and Methods

### Study Participants

This prospective cross-sectional study involved women who attended the postpartum rehabilitation clinic at the First Affiliated Hospital of Gannan Medical University between May 2022 and September 2023. Data collection occurred within 6–8 weeks postpartum. All patients' cesarean section scars were assessed by the same evaluator to ensure consistency in the evaluations. Women with severe organic organ disease, history of prior abdominal surgery, or inability to provide accurate responses were excluded. All patients provided written informed consent. The study was approved by the Ethics Committee of the First Affiliated Hospital of Gannan Medical University (approval number LLSC-2022042802) and was registered in the Chinese Clinical Trial Registry (registration number: ChiCTR2200059785; date of enrollment: 11 May 2022).

### Scar

All patients' cesarean section scars were assessed by the same evaluator to ensure consistency in the evaluations. Jinglong and Zhehu pointed out that from a pathological perspective, skin scars are classified into two main categories: normal scars (physiological scars) and abnormal scars (pathological scars). The latter primarily includes hypertrophic scars and keloids. Normal scars are characterized by rapid maturation, no contraction, no increase in width, and collagen formation that is sufficient to maintain strength. They do not protrude above the surface of the tissue and have a normal or near-normal color. If a scar initially appears raised above the skin surface but does not exceed the area of tissue damage, and gradually flattens out over time with symptoms of itching and discomfort subsiding, it is classified as a hypertrophic scar. Conversely, if the scar remains raised above the skin surface, continues to grow, and exceeds the area of tissue damage without flattening on its own—especially if it recurs after simple surgical removal and is larger than the original lesion—this is classified as a keloid [9]. During the data collection process, we did not encounter any patients with keloids. Therefore, we categorized the scars in this study into three types: no scar, normal scar, and hypertrophic scar.

### Active Pelvic Floor Muscle Tone

We assessed the active pelvic floor muscle tone and strength in postpartum women using the Glazer protocol. The participants were positioned supine with their upper body at a 120° angle to their lower limbs, and their hips and knees slightly flexed, while maintaining a relaxed overall posture. The vaginal electrode was inserted into the vaginal tube at a depth of 3 to 4 cm, with its metal plate positioned at the 3 to 9 o'clock position. Additionally, a reference electrode was placed on the anterior–superior iliac spine. The participants were instructed by the software to contract and relax their pelvic floor muscles continuously. During pelvic floor muscle contraction, efforts were made to minimize the involvement of the abdominal, gluteal, and thigh adductor muscles.

**Fig. 1:**
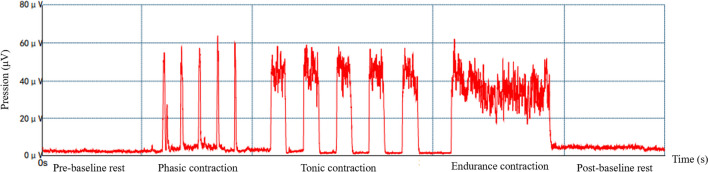
Schematic diagram of the Glazer protocol

The specific steps are outlined as shown in Fig. 1. During the pre-baseline rest period the active pelvic floor muscle tension is measured under a completely relaxed state for 60 s, including the collection of its average electromyography (EMG) value and variability. The rapid contraction phase assesses the capacity for rapid muscle contraction and recovery. Data collection includes the maximum EMG value, rise time, and fall time. The sustained contraction period consists of continuous muscle contraction for 10 s to collect the average EMG value and variability of sustained muscle contraction. During the endurance contraction stage the endurance of slow muscle contraction is assessed, with the collection of the average EMG value and variability. The post-test resting phase is similar to the pre-test resting state. This phase reflects the pelvic floor muscle active tension in a fully relaxed state for 60 s and involves the collection of its mean EMG value and variability [7, 13]. The Glazer protocol was performed using a pelvic floor muscle sEMG device (MLD A2; Medlander, Nanjing, China).

### Covariates

We included the following variables: age, height, body weight, body mass index (BMI), weight gained during pregnancy, number of pregnancies, primiparous or multiparous, parity, birth weight, gestational age, mode of delivery (vaginal delivery or cesarean section), hypertension [14], type 2 diabetes mellitus (T2DM) [15], thalassemia [16], feeding mode (breast, formula, or mixed feeding), data from intrapelvic sEMG assessment using the Glazer protocol [7]. The selection of these variables is based on their clinical relevance, findings from previous literature, significant covariates identified in univariate regression analysis, and variables for which the change in effect estimates exceeded 10%.

### Statistical Analysis

Histogram distribution was employed to assess the normality of the variables. Normally distributed continuous variables were reported as mean ± SD, whereas skewed continuous variables were described as median (interquartile range [IQR]). Categorical variables were presented as frequencies (%). We utilized Chi-squared or Fisher's exact tests for categorical variables, one-way analysis of variance for normally distributed variables, and the Kruskal–Wallis *H* test for skewed distributions to assess differences among the various scar groups. The Bonferroni correction least significant difference method was applied for multiple comparisons.

The effect of cesarean section scars on active pelvic floor muscle tone was evaluated using linear regression models (regression coefficients β and 95% confidence interval [CI]) with adjustment for major covariables. The selection of these confounders was based on clinical relevance, previous literature, all significant covariates in the univariate analysis, or their associations with the outcomes of interest, or a change in effect estimate exceeding 10%. Potential multicollinearity was assessed using the variance inflation factor (VIF), with an VIF ≥ 5 indicating the presence of multicollinearity. We constructed three models. Model 1 adjusted for age, height, weight, and BMI. Model 2 additionally adjusted for weight gained during pregnancy, number of pregnancies, parity, multiparas, birth weight, and gestational age. Model 3 further adjusted for hypertension, type 2 diabetes, thalassemia, and feeding method.

We employed multiple imputation, based on five replications and a chained equation approach method, to maximize statistical power and minimize bias that may result from missing data [17]. Furthermore, in order to evaluate the robustness of the findings, we conducted a series of sensitivity analyses by combining the normal scar group and the hypertrophic scar group, and then compared them with the no scar group. Within the sensitivity analysis, we applied several additional association inference models, such as propensity score adjustment [18], propensity score matching (PSM) [19], treatment-weighted inverse probability [20], standardized mortality weighting [21], pairwise algorithmic, and overlap weight [22]. All analyses were conducted using the statistical software packages R (http://www.R-project.org, R Foundation) and Free Statistics software version 1.7.1. All statistical tests were two tailed, and statistical significance was defined as *p* < 0.05.

## Results

### Participants’ Characteristics

**Fig. 2:**
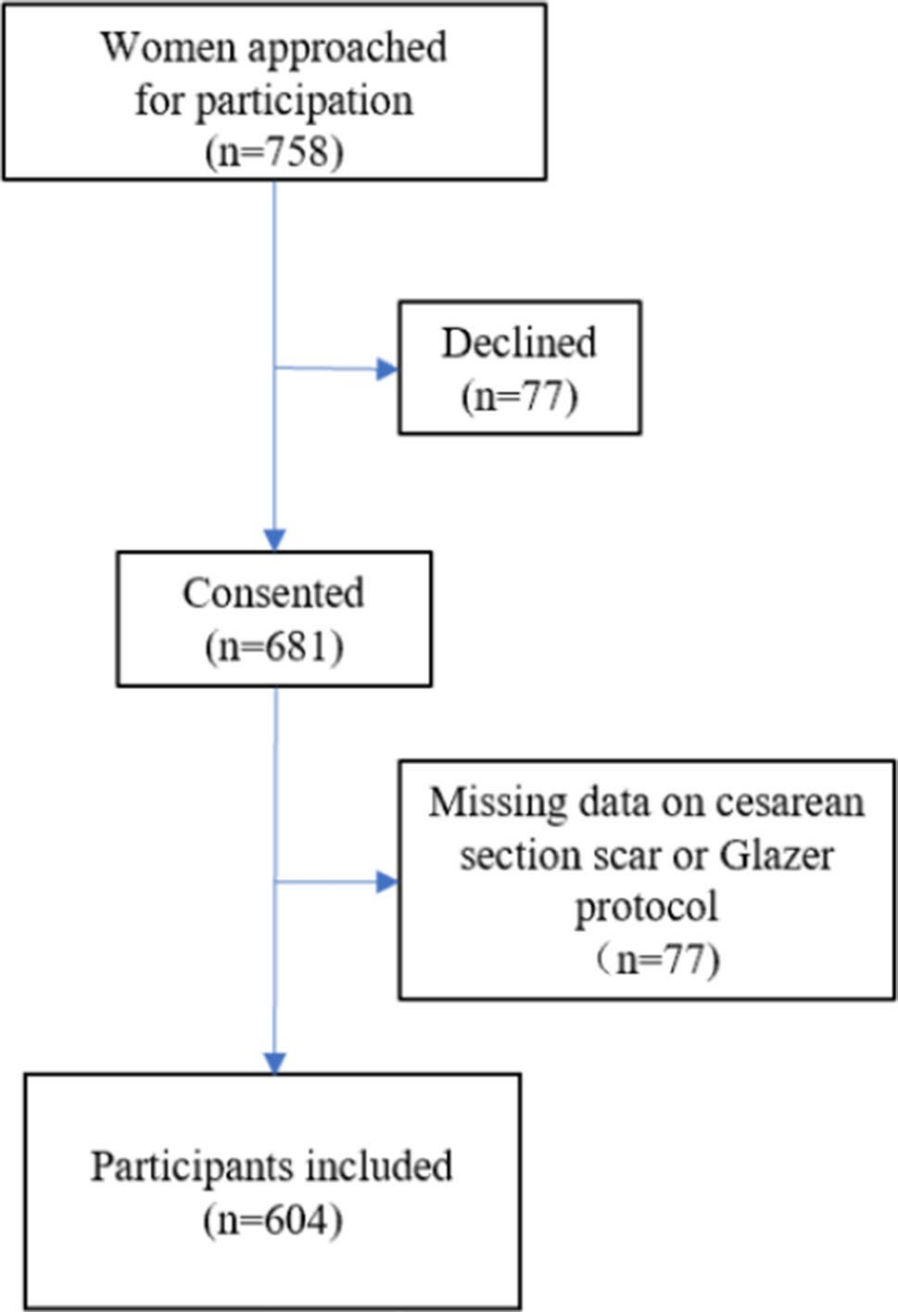
The flow chart of the study

Out of 758 women who visited the postnatal rehabilitation department 6 weeks after delivery, 681 (89.8%) consented to be interviewed, and data on active pelvic floor muscle tone and scarring were available for 604 (79.7%) participants. Among the 604 participants, 445 (73.7%) had no cesarean section scars, 133 (22.0%) had normal scars, and 26 (4.3%) had hypertrophic scars (Fig. 2). There were no participants with keloid scars in this study. The clinical characteristics of the three groups are detailed in Table 1. The mean age of all participants was 27.7 ± 4.5 years. The average amplitudes of pre-baseline rest for the no scar, normal scar, and hypertrophic scar groups were 4.4 (2.8, 6.1), 5.9 (2.9, 7.8), and 9.1 (7.1, 11.2) respectively. Similarly, the average amplitudes of post-baseline rest were 3.6 (2.1, 5.5), 4.4 (2.4, 6.6), and 9.0 (7.3, 10.8) respectively.

**Table 1:** Baseline characteristics of the participants

Variables	Total (*n* = 604)	No scar (*n* = 445)	Normal scar (*n* = 133)	Hypertrophic scar (*n* = 26)
Age (years)	27.7 ± 4.5	27.3 ± 4.4	29.0 ± 4.7	29.5 ± 4.8
Height (cm)	158.5 ± 4.9	158.6 ± 4.7	157.8 ± 5.4	158.7 ± 4.2
Body weight (kg)	58.2 ± 7.8	58.0 ± 7.5	59.0 ± 8.9	58.0 ± 6.5
BMI (kg/m^2^)	23.2 ± 2.8	23.1 ± 2.7	23.6 ± 3.1	22.9 ± 2.3
Weight gained during pregnancy (kg)	14.7 ± 4.8	14.7 ± 4.7	14.8 ± 5.2	13.7 ± 4.2
Number of pregnancies	2.0 (1.0, 3.0)	2.0 (1.0, 3.0)	2.0 (1.0, 3.0)	2.0 (1.0, 2.8)
Parity	1.7 ± 0.8	1.6 ± 0.8	1.7 ± 0.8	1.7 ± 0.6
Multiparous, *n* (%)				
No	316 (52.3)	247 (55.5)	59 (44.4)	10 (38.5)
Yes	288 (47.7)	198 (44.5)	74 (55.6)	16 (61.5)
Birth weight (kg)	3.3 ± 0.5	3.3 ± 0.4	3.3 ± 0.6	3.2 ± 0.8
Gestational age (weeks)	39.2 ± 1.4	39.3 ± 1.3	38.8 ± 1.5	38.5 ± 1.7
Mode of delivery, *n* (%)				
Vaginal delivery	449 (74.3)	445 (100)	2 (1.5)	2 (7.7)
Cesarean delivery	155 (25.7)	0 (0)	131 (98.5)	24 (92.3)
Hypertension, *n* (%)				
No	563 (95.6)	421 (97)	119 (91.5)	23 (92)
Yes	26 (4.4)	13 (3)	11 (8.5)	2 (8)
T2DM, *n* (%)				
No	521 (87.7)	390 (89)	108 (82.4)	23 (92)
Yes	73 (12.3)	48 (11)	23 (17.6)	2 (8)
Thalassemia, *n* (%)				
No	529 (90.3)	379 (87.9)	125 (96.2)	25 (100)
Yes	57 (9.7)	52 (12.1)	5 (3.8)	0 (0)
Feeding method, *n* (%)				
Breast feeding	278 (57.7)	217 (60.3)	52 (50)	9 (50)
Formula feeding	45 ( 9.3)	30 (8.3)	13 (12.5)	2 (11.1)
Mixed feeding	159 (33.0)	113 (31.4)	39 (37.5)	7 (38.9)
Glazer assessment				
Pre-baseline rest				
Average amplitude (μV)	4.7 (2.9, 6.9)	4.4 (2.8, 6.1)	5.9 (2.9, 7.8)	9.1 (7.1, 11.2)
Variation coefficient (%)	0.2 (0.1, 0.2)	0.2 (0.1, 0.2)	0.2 (0.1, 0.2)	0.1 (0.1, 0.2)
Rapid contraction				
Maximal values (μV)	30.4 (22.2, 40.0)	29.1 (20.7, 38.2)	34.8 (27.3, 46.8)	30.1 (26.2, 33.6)
Rise time (s)	0.5 (0.4, 0.6)	0.5 (0.4, 0.6)	0.5 (0.4, 0.6)	0.5 (0.4, 0.7)
Recovery time (s)	0.6 (0.4, 0.8)	0.6 (0.4, 0.8)	0.6 (0.4, 0.8)	0.6 (0.5, 0.9)
Sustained contraction				
Average amplitude (μV)	19.9 (13.7, 27.7)	18.6 (13.1, 26.4)	23.6 (17.3, 31.2)	21.6 (15.7, 27.4)
Variation coefficient (%)	0.2 (0.2, 0.3)	0.2 (0.2, 0.3)	0.2 (0.2, 0.3)	0.2 (0.2, 0.3)
Endurance contraction				
Average amplitude (μV)	17.4 (12.4, 24.7)	16.1 (11.5, 22.8)	22.1 (16.4, 28.3)	21.9 (14.7, 25.1)
Variation coefficient (%)	0.2 (0.2, 0.3)	0.2 (0.2, 0.3)	0.2 (0.2, 0.3)	0.2 (0.2, 0.2)
Post-baseline rest				
Average amplitude (μV)	3.9 (2.2, 6.2)	3.6 (2.1, 5.5)	4.4 (2.4, 6.6)	9.0 (7.3, 10.8)
Variation coefficient (%)	0.2 (0.1, 0.2)	0.2 (0.1, 0.2)	0.2 (0.1, 0.2)	0.2 (0.1, 0.2)

*BMI* body mass index, *T2DM* type 2 diabetes mellitus

### Relationship Between Cesarean Section Scars and Active Pelvic Floor Muscle Tone

In the univariate linear regression analysis, a positive correlation was observed between normal scar and the average amplitudes of pre-baseline rest (β = 1.25, 95% CI = 0.69–1.81, *p* < 0.001). The positive correlation between hypertrophic scars and average amplitudes of pre-baseline rest was even higher (β = 4.7, 95% CI = 3.56–5.84, *p* < 0.001). Similarly, normal scar correlated positively with average amplitudes of post-baseline rest (β = 1.03, 95% CI = 0.48–1.59, *p* < 0.001), whereas hypertrophic scars exhibited an even higher positive correlation with average amplitudes of post-baseline rest (β = 4.8, 95% CI = 3.67–5.92, *p* < 0.001). The associations were strengthened and became statistically significant (β = 1.68, 5.09, 1.47, 5.03, 95% CI = 1.02–2.33, 3.69–6.5, 0.8–2.15, 3.6–6.47 respectively, all *p* < 0.001), independent of potential confounders (Table 2, model 3). In the multiple imputation model, the results remained stable (β = 1.39, 4.87, 1.08, 4.93, 95% CI = 0.81–1.96, 3.73–6.02, 0.51–1.65, 3.79–6.07 respectively, all *p* < 0.001; Table 2, post-imputation).

**Table 2:** Multiple linear regression analysis of the relationship between cesarean section scar and pelvic floor muscle tone in women at 6–8 weeks postpartum

Variables	No scar (*n* = 445)	Normal scar (*n* = 133)	Hypertrophic scar (*n* = 26)
	β (95% CI)	β (95% CI)	β (95% CI)
Average amplitude of pre-baseline rest			
Unadjusted	0 (Ref)	1.25 (0.69~1.81)	4.70 (3.56~5.84)
Model 1	0 (Ref)	1.35 (0.78~1.91)	4.97 (3.81~6.13)
Model 2	0 (Ref)	1.30 (0.72~1.87)	5.09 (3.90~6.29)
Model 3	0 (Ref)	1.68 (1.02~2.33)	5.09 (3.69~6.50)
Post-imputation	0 (Ref)	1.39 (0.81~1.96)	4.87 (3.73~6.02)
Average amplitude of post-baseline rest			
Unadjusted	0 (Ref)	1.03 (0.48~1.59)	4.8 (3.67~5.92)
Model 1	0 (Ref)	1.10 (0.54~1.66)	4.97 (3.83~6.12)
Model 2	0 (Ref)	1.09 (0.52~1.66)	5.20 (4.02~6.38)
Model 3	0 (Ref)	1.47 (0.80~2.15)	5.03 (3.60~6.47)
Post-imputation	0 (Ref)	1.08 (0.51~1.65)	4.93 (3.79~6.07)

Adjusted covariates: unadjusted = scar; model 1 = unadjusted + (age, height, weight, BMI); model 2 = model 1 + (weight gained during pregnancy, number of pregnancies, multiparas, birth weight, gestational age); model 3 = model 2 + (hypertension, T2DM, thalassemia, feeding method)

*CI* confidence interval, *BMI* body mass index, *T2DM* type 2 diabetes mellitus

Additionally, PSM analysis was conducted to adjust for the primary confounding covariates between the cesarean section scar group and the no scar group, in order to assess the consistency of our results. Similar results were obtained even after adjusting for multiple factors (Appendix A and B).

## Discussion

Studies have shown that age and vaginal delivery are risk factors for postpartum organ prolapse (POP) and postpartum urinary incontinence (UI), whereas BMI is specifically a risk factor for postpartum UI. Additionally, fetal birth weight is identified as a risk factor for POP [23]. Yang and Liao found that obstetric factors, such as age, mode of delivery, and the presence of a large fetus, may increase a woman's risk of developing pelvic floor disorders in the early postpartum period. They proposed that these risk factors should be accurately identified and promptly addressed to prevent the development of pelvic floor disorders [24]. In this study, we selected variables such as age, BMI, weight gained during pregnancy, parity, mode of delivery, birth weight, gestational age, feeding mode, and complications. The selection of these variables was based on their clinical relevance, the published literature, significant covariates identified in univariate regression analysis, and variables where the change in effect estimates exceeded 10%.

In this cross-sectional study, we assessed active pelvic floor muscle tone by measuring the average amplitudes of pre-baseline rest and post-baseline rest. Our findings revealed that cesarean section scar severity is positively associated with increased active pelvic floor muscle tone in women at 6–8 weeks postpartum (Table 2, model 3). Notably, this result remained robust in propensity score analysis (Appendix A and B). Furthermore, we observed that women with hypertrophic scars exhibited higher active pelvic floor muscle tone than those with normal scars, with this outcome persisting after multiple interpolations.

Many women choose cesarean section to avoid the pain associated with vaginal delivery, pelvic floor and vaginal trauma, gynecological examination, and prolonged labor [25]. However, women who undergo cesarean delivery face an increased risk of chronic pelvic pain [1, 26, 27]. It is important to note that the exact mechanisms behind this phenomenon are not yet fully understood. The findings of this study suggest that the severity of cesarean section scars might correlate positively with an increase in active pelvic floor muscle tone. The underlying biological mechanism may involve scar tissue formed after the cesarean section causing the uterus to adhere to the abdominal wall, which in turn increases active tension in the pelvic floor muscles. This aligns with the observations of Hardy and Rousseau, who propose that the tension and adhesions resulting from cesarean delivery can create a connection between the uterus and the abdominal wall, potentially triggering severe chronic pelvic pain [4]. We hypothesize that scar tissue formed after a cesarean section might cause chronic inflammatory responses and fibrotic processes, leading to adhesions within the pelvic cavity. This could alter the position of internal organs, increase pelvic active tension, and potentially affect pelvic floor muscle function or contribute to chronic pelvic pain. Moving forward, we will continue to explore the relationship between chronic pelvic pain, cesarean section scars, and active pelvic floor muscle tone, aiming to provide more valuable information for clinical practice. Furthermore, to gain a comprehensive understanding of the relationship between internal scars and skin surface scars (especially hypertrophic scars and keloids), we will consider incorporating imaging studies (such as ultrasound or nuclear magnetic resonance) to examine the relationship between postoperative skin scars and internal tissue adhesions, and analyze the correlation between the two.

Our findings are robust for several reasons. First, we had a relatively large sample cohort (*n* = 604). Additionally, the variables we gathered were comprehensive, encompassing not only clinical characteristics but also feeding patterns, concurrent comorbidities, and Glazer assessment parameters. However, there are certain limitations to this study. First, as a cross-sectional observational study, the associations identified may not imply direct causal relationships. Second, the study was performed at a single center. Third, factors other than the muscles themselves, such as adipose tissue and vaginal lubrication, may inevitably influence microvolt levels. Fourth, the sample size in the hypertrophic scar group of this study was relatively small. Finally, factors influencing the generalizability of the findings include the absence of participation from all age groups of women. Our study focused on women of childbearing age 6–8 weeks postpartum, excluding postmenopausal women. Hence, caution should be exercised when extrapolating these results to older women.

## Conclusion

In summary, this study demonstrates a significant positive correlation between the severity of cesarean section scars and increased active pelvic floor muscle tone. This finding provides important information for clinicians when developing personalized rehabilitation plans. Preventing and treating cesarean section scars may help to reduce active pelvic floor muscle tension and improve recovery outcomes and quality of life for postpartum women.

## Supplementary Information

Below is the link to the electronic supplementary material.

Supplementary file1 (DOCX 16 KB)

Supplementary file2 (DOCX 16 KB)

Supplementary file3 (DOCX 17 KB)

Supplementary file4 (DOCX 17 KB)
