# Supplementary material for: Association Between Cesarean Scar and Pelvic Floor Muscle Tone at 6–8 Weeks Postpartum
Source: Int Urogynecol J. 2025 Jan 9;36(3):607–13. doi: 10.1007/s00192-024-06023-8 (PMC12003483; doi:10.1007/s00192-024-06023-8)
Supplement: Supplementary file 1 — Supplementary file1 (DOCX 16 KB) [file 192_2024_6023_MOESM1_ESM.docx]

Appendix A

Table 3 Associations between cesarean section scar and the average amplitudes of pre-baseline rest in women at 6-8 weeks postpartum in propensity score analyses

| Analysis | *β* (95% *CI*) | *p* value |
| --- | --- | --- |
| Univariate regression analysis | 2.07 (1.46-2.68) | < 0.001 |
| Multivariate regression analysis | 2.16 (1.53-2.80) | < 0.001 |
| With PSA | 2.17 (1.52-2.82) | < 0.001 |
| With PSM | 2.04 (1.17-2.92) | < 0.001 |
| With IPTW | 2.28 (1.67-2.89) | < 0.001 |
| With SMRW | 1.96 (1.32-2.59) | < 0.001 |
| With PA | 2.08 (1.50-2.66) | < 0.001 |
| With Ow | 2.17 (1.59-2.74) | < 0.001 |

*CI* confidence interval, *PSA* propensity score adjustment, *PSM* propensity score matching, *IPTW* inverse probability weighting, *SMRW* standardized mortality weighting, *PA* pairwise algorithmic, *OW* overlap weight
