# Supplementary material for: Association Between Cesarean Scar and Pelvic Floor Muscle Tone at 6–8 Weeks Postpartum
Source: Int Urogynecol J. 2025 Jan 9;36(3):607–13. doi: 10.1007/s00192-024-06023-8 (PMC12003483; doi:10.1007/s00192-024-06023-8)
Supplement: Supplementary file 2 — Supplementary file2 (DOCX 16 KB) [file 192_2024_6023_MOESM2_ESM.docx]

Appendix B

Table 4 Associations between cesarean section scar and the average amplitudes of post-baseline rest in women at 6-8 weeks postpartum in propensity score analyses

| Analysis | *β* (95% CI) | *p* value |
| --- | --- | --- |
| Univariate regression analysis | 1.89 (1.27-2.51) | < 0.001 |
| Multivariate regression analysis | 1.98 (1.32-2.63) | < 0.001 |
| With PSA | 1.98 (1.32-2.64) | < 0.001 |
| With PSM | 1.57 (0.65-2.50) | < 0.001 |
| With IPTW | 2.08 (1.45-2.71) | < 0.001 |
| With SMRW | 1.78 (1.12-2.44) | < 0.001 |
| With PA | 1.88 (1.26-2.51) | < 0.001 |
| With Ow | 1.98 (1.37-2.60) | < 0.001 |

*CI confidence interval, PSA propensity score adjustment, PSM propensity score matching, IPTW inverse probability weighting, SMRW standardized mortality weighting, PA pairwise algorithmic, OW overlap weight*
