# Supplementary material for: Association Between Cesarean Scar and Pelvic Floor Muscle Tone at 6–8 Weeks Postpartum
Source: Int Urogynecol J. 2025 Jan 9;36(3):607–13. doi: 10.1007/s00192-024-06023-8 (PMC12003483; doi:10.1007/s00192-024-06023-8)
Supplement: Supplementary file 3 — Supplementary file3 (DOCX 17 KB) [file 192_2024_6023_MOESM3_ESM.docx]

Appendix C

Table 5 Univariable linear regression analysis of the relationship between variables and average amplitude of pre-baseline rest in women at 6-8 weeks postpartum

| Variables | *β* (95% CI) | *P* value |
| --- | --- | --- |
| Age (years) | -0.03 (-0.08,0.03) | 0.325 |
| Height (cm) | -0.03 (-0.08,0.02) | 0.27 |
| BMI (kg/m^2^ ) | 0.06 (-0.03,0.15) | 0.177 |
| Body weight (kg) | 0.01 (-0.02,0.04) | 0.559 |
| Weight gained during pregnancy (kg) | 0.04 (-0.01,0.09) | 0.15 |
| Number of pregnancies | 0.02 (-0.17,0.21) | 0.839 |
| Parity | 0.2 (-0.11,0.5) | 0.204 |
| Multiparous vs primiparous | 0.2 (-0.29,0.69) | 0.429 |
| Birth weight (kg) | -0.6 (-1.09,-0.11) | 0.017 |
| Gestational age (weeks) | -0.2 (-0.38,-0.03) | 0.025 |
| Cesarean delivery vs Vaginal delivery | 1.77 (1.23,2.32) | < 0.001 |
| No scar: ref.=0 |  |  |
| Normal scar | 1.25 (0.69,1.81) | < 0.001 |
| Hypertrophic scar | 4.7 (3.56,5.85) | < 0.001 |
| Hypertension | 0.85 (-0.37,2.06) | 0.171 |
| T2DM | -0.22 (-0.97,0.54) | 0.576 |
| Thalassemia | -0.36 (-1.21,0.49) | 0.405 |
| Breast feeding: ref.=0 |  |  |
| Formula feeding | -0.62 (-1.58,0.34) | 0.203 |
| Mixed feeding | -0.04 (-0.63,0.56) | 0.903 |

*CI* confidence interval, *BMI* body mass index, *T2DM* type 2 diabetes mellitus
