# Supplementary material for: Association Between Cesarean Scar and Pelvic Floor Muscle Tone at 6–8 Weeks Postpartum
Source: Int Urogynecol J. 2025 Jan 9;36(3):607–13. doi: 10.1007/s00192-024-06023-8 (PMC12003483; doi:10.1007/s00192-024-06023-8)
Supplement: Supplementary file 4 — Supplementary file4 (DOCX 17 KB) [file 192_2024_6023_MOESM4_ESM.docx]

Appendix D

Table 6 Univariable linear regression analysis of the relationship between variables and average amplitude of post-baseline rest in women at 6-8 weeks postpartum

| Variables | *β* (95% CI) | *P* value |
| --- | --- | --- |
| Age (years) | 0 (-0.05,0.05) | 0.992 |
| Height (cm) | -0.03 (-0.08,0.02) | 0.186 |
| BMI (kg/m^2^) | 0.02 (-0.06,0.11) | 0.584 |
| Body weight (kg) | 0 (-0.04,0.03) | 0.812 |
| Weight gained during pregnancy (kg) | 0.02 (-0.03,0.07) | 0.483 |
| Number of pregnancies | 0.11 (-0.08,0.3) | 0.26 |
| Parity | 0.33 (0.03,0.63) | 0.029 |
| Multiparous vs primiparous | 0.31 (-0.18,0.8) | 0.214 |
| Birth weight (kg) | -0.49 (-0.98,0) | 0.048 |
| Gestational age (weeks) | -0.17 (-0.34,0) | 0.057 |
| Cesarean delivery vs Vaginal delivery | 1.64 (1.1,2.18) | < 0.001 |
| No scar: ref.=0 |  |  |
| Normal scar | 1.03 (0.48,1.59) | < 0.001 |
| Hypertrophic scar | 4.8 (3.67,5.92) | < 0.001 |
| Hypertension | 0.75 (-0.46,1.97) | 0.225 |
| T2DM | -0.21 (-0.96,0.53) | 0.573 |
| Thalassemia | -0.36 (-1.2,0.47) | 0.394 |
| Breast feeding: ref.=0 |  |  |
| Formula feeding | -0.5 (-1.49,0.49) | 0.32 |
| Mixed feeding | -0.25 (-0.85,0.36) | 0.424 |

*CI* confidence interval*, BMI* body mass index*, T2DM* type 2 diabetes mellitus
